# Gastrinoma in multiple endocrine neoplasia type 1 after total pancreatectomy: A case report

**DOI:** 10.1097/MD.0000000000018275

**Published:** 2019-12-16

**Authors:** Shu Gong, Zhi Li, Xu-Bao Liu, Xin Wang, Wen-Wu Shen

**Affiliations:** a Department of Pancreatic Surgery; b Outpatient Department, West China Hospital, Sichuan University, Chengdu, Sichuan Province, China.

**Keywords:** gastrinoma, MEN-1, total pancreatectomy

## Abstract

**Rationale:**

Surgery for patients with multiple endocrine neoplasia type 1(MEN-1) related gastrinoma remains controversial and total pancreatectomy (TP) has rarely been performed. We reported a case of patient with MEN-1 related gastrinoma treated by TP.

**Patient concerns:**

A 46-year-old female was admitted to our hospital due to abdominal distension and diarrhea for 2 years. The patient underwent pituitary tumor resection and kidney stone lithotripsy 10 years ago.

**Diagnoses:**

Abdominal computed tomography showed single lesion in the duodenum and multiple lesions throughout the pancreas. The patient's gastrin level was significantly increased (1080 pg/ml). These findings in combination with the pituitary tumor history suggested the presence of gastrinoma associated with MEN-1 syndrome.

**Intervention:**

An exploratory laparotomy was performed. Intraoperative ultrasound confirmed the numerous tumors diffusely distributed throughout the pancreas and the patient eventually underwent TP.

**Outcomes:**

Twelve months later, the patient was hospitalized again for anastomotic fistula and underwent a partial gastrectomy, small bowel resection and drainage of the abscess. One month later, she received gastrostomy and jejunostomy due to digestive tract fistula, and died a month later (14 months after TP).

**Lessons:**

There still might be the possibility of recurrence even after radical surgical resection of gastrinomas, and we suggest the need to measure the basal acid output and maintain regular anti-acid therapy in the long-term follow-up of patients with MEN-1 related gastrinoma.

## 1 Introduction

Gastrinoma is the second most common functional pancreatic neuroendocrine tumor (pNET), with a yearly incidence of approximately 0.5 to 21.5 cases per a million of people worldwide.^[1]^ Gastrinomas are located predominantly in the duodenum (70%) and pancreas (25%). They are characterized by gastric hypersecretion that results in peptic ulcers and diarrhea; this condition is known as Zollinger–Ellison syndrome (ZES).^[1–4]^ Most gastrinomas are sporadic (75%–80%), whereas approximately 20% to 25% are associated with multiple endocrine neoplasia type 1(MEN-1).^[1]^ In patients with MEN-1, tumors are generally small, multiple and have a high tendency to metastasize; thus, achieving biochemical cure without radical surgical resection is impossible.^[6,7]^ However, surgery for patients with MEN-1 remains controversial because of its potential short- and long-term complications,^[4–7]^ and a total pancreatectomy (TP) has rarely been performed. Here, we reported a case of a patient with MEN-1 associated gastrinoma treated by TP.

## 2 Case presentation

**Figure 1:**
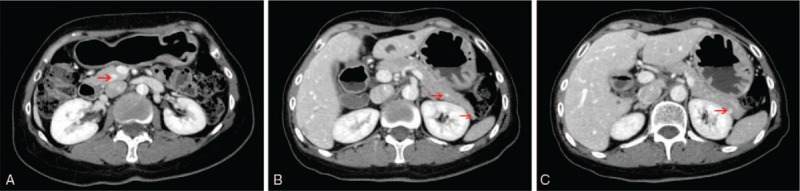
Preoperative abdominal computed tomographic (CT) scan. A: One local thickening in the descending duodenum wall (red arrows); B: Several lesions located in the head, body and tail of pancreas (red arrows).

A 46-year-old female was admitted to our hospital complaining of abdominal distension and diarrhea for 2 years. Gastroscopy revealed duodenal ulcer, and she was treated with proton pump inhibitors (PPIs) for half a year, but symptoms persisted. The patient underwent pituitary tumor resection and kidney stone lithotripsy 10 years ago. The patient's gastrin level was significantly elevated (1080 pg/ml; normal range: 5–100 pg/ml). Parathyroid hormone, blood calcium, and serum tumor markers (AFP, CEA, CA 19–9, and CA 125) were normal. Abdominal computed tomography (CT) showed one local thickening of descending duodenum wall and several lesions located in the head, body, and tail of pancreas, and the largest one, which measured 0.8 cm × 0.6 cm, was located in the pancreatic head (Fig. 1). These findings in combination with the pituitary tumor history suggested the presence of gastrinoma associated with MEN-1 syndrome.

**Figure 2:**
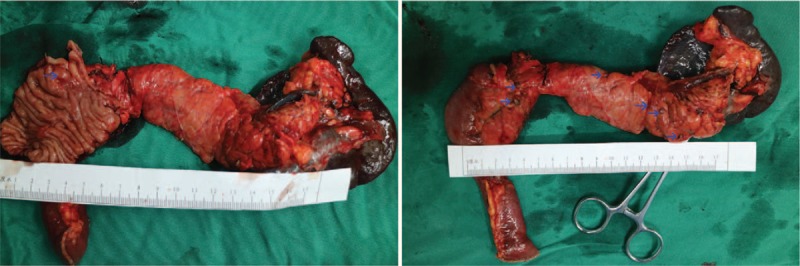
Macroscopic appearance of the resection specimen. A: A tumor was found in the duodenum (blue arrows). B: Several tumors were found in the head, body and tail of the pancreas (blue arrows).

An exploratory laparotomy was performed. In surgery, several tumors were found in the head, body, and tail of pancreas; furthermore, a tumor without well-defined boarders was palpable in the duodenum (Fig. 2). Intraoperative ultrasound confirmed that numerous tumors were diffusely distributed throughout the pancreas. The patient eventually underwent TP with peripancreatic lymph node resection.

**Figure 3:**
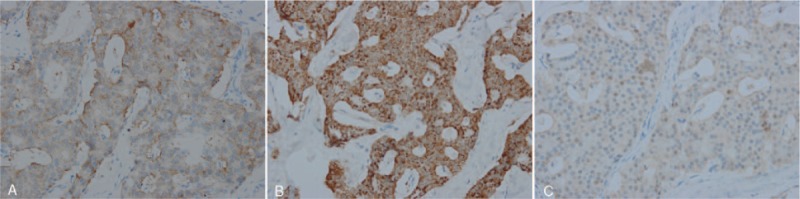
Immunohistochemical stains (×400). Tumor cells were positive for Syn (A), CgA (B), and gastrin (C).

Pathology confirmed a multifocal neuroendocrine neoplasm, of which 1 gray solid nodule measuring 1 cm × 0.5 cm × 0.5 cm was found in the submucosa of the descending duodenum; 12 gray solid nodules were found in the head, body, and tail of the pancreas, ranging from 0.3 cm to 0.8 cm in diameter. Additionally, one was classified as grade 2 (Ki-67 index, 3%–5%), and the others were grade 1 (Ki-67 index, <2%) according to the World Health Organization 2010 classification system. Immunohistochemical analysis results revealed that the tumor cells were positive for Syn, CgA, and gastrin (Fig. 3). Moreover, two metastatic peripancreatic lymph nodes were identified (Ki-67 index, <2%).

**Figure 4:**
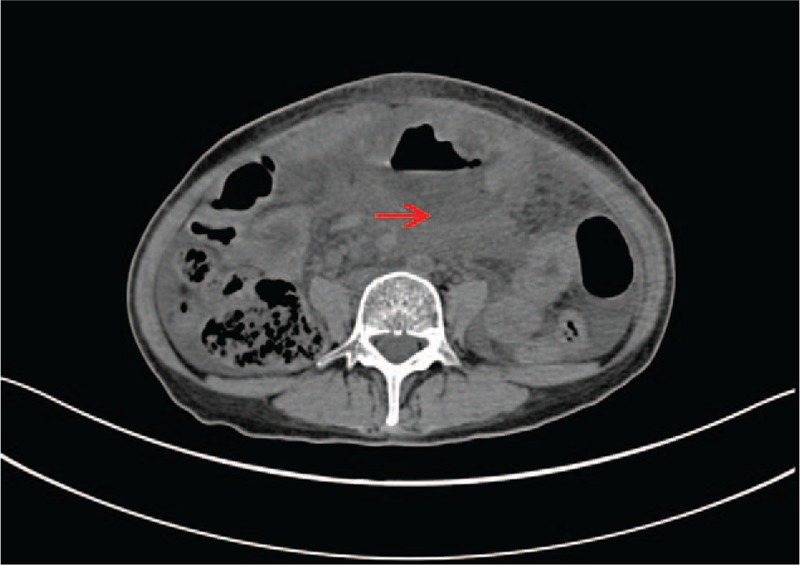
Abdominal CT scan 12 months after TP. It showed encapsulated effusion and gases surrounding the gastrointestinal anastomosis (red arrows).

Postoperative gastrin level decreased to 23.25 pg/ml. The patient recovered uneventfully and was discharged 7 days after surgery. After discharge, the patient persisted regular follow-up in endocrine clinic and had a stable control of blood glucose. Twelve months later, she was hospitalized for progressive abdominal pain with fullness and fever. The gastrin level was almost within normal limits (125 pg/ml). CT scan revealed encapsulated effusion and gases surrounding the gastrointestinal anastomosis (Fig. 4), thereby suggesting an anastomotic fistula. Then, a laparotomy was performed again, and a huge abscess measuring 5 cm × 10 cm was found around the anastomosis. The patient underwent partial gastrectomy, small bowel resection, and drainage of the abscess. One month later, the patient was hospitalized again for digestive tract fistula. Gastrostomy and jejunostomy were performed, and the patient died for severe malnutrition 1 month later (14 months after TP).

Informed consent was obtained from the patient's son for this publication and any accompanying images.

## 3 Discussion

MEN-1 is a rare, autosomal dominant inherited syndrome caused by mutations in the MEN-1 tumor suppressor gene. Patients can be diagnosed with MEN-1 when 2 or more primary endocrine tumors, including pituitary tumors, pancreatic tumors, and parathyroid adenomas, associated with MEN-1 are present.^[8]^ The incidence of MEN-1 is 0.25% and 16% to 38% in patients with gastrinomas.^[8]^ In patients with MEN-1 associated gastrinomas, the average onset is 5 to 10 years earlier than that of sporadic gastrinomas and generally before 50 years old.^[9,10]^ Gastrinomas in patients with MEN-1 mostly occur in the duodenum (85%–100%) and less commonly in the pancreas (0%–15%); the tumors are often multiple and small (<0.5 cm) and are associated with lymph node metastases in 40% to 60% of the cases.^[4,11]^ In our case, interestingly, tumors were mainly located in the pancreas.

The most common presenting symptoms of gastrinomas include abdominal pain, diarrhea, and esophageal symptoms.^[2]^ The diagnostic evaluation of gastrinomas begins by measuring a fasting serum concentration of gastrin. If the fasting serum gastrin is more than10-fold normal (<100 pg/ml) and the gastric pH is < 2, a diagnosis of gastrinoma is established.^[1]^ Ultrasonography, CT, MRI, somatostatin receptor scintigraphy, and endoscopic ultrasonography are useful and important modalities for localization diagnosis and ruling out metastases.^[5,7,12,13]^

Surgery for patients with MEN-1 associated gastrinomas remains controversial. Several authors conclude that the multicentricity of tumors mostly in the duodenum preclude surgical cure and suggest medical therapy, such as PPIs and/or somatostatin analogs.^[14,15]^ Others recommend surgical resection of tumors only if the tumor is larger than 2 to 3 cm, and enucleation at surgery remains the generally recommended surgical procedure.^[16,17]^ However, larger tumors are often associated with increased metastatic risk and, consequently, reduced curability.^[17]^ Therefore, some surgeons support a more aggressive surgery, such as pancreas-preserving total duodenectomy, Whipple pancreaticoduodenectomy, or TP, to reduce the risk of distant metastases, achieve biochemical cure, and improve survival.^[18–20]^

There is a general agreement that TP should be avoided, except in cases of multiple and diffuse macroscopic lesions. To date, TP has rarely been performed for patients with MEN-1. We searched literature and found only 3 cases^[20,21]^ of MEN-1 receiving TP as initial surgery because of the diffuse presence of macroscopic tumors within the pancreas; furthermore, 3 cases^[20,22]^ had TP at reoperation, of which^[20]^ TP in one case was as a rescue after the dehiscence of pancreato-jejunal anastomosis. However, the long-term survival of the above cases had been seldom reported. In our case, the patient died 14 months later from serious infection and severe malnutrition caused by the disruption of gastrointestinal anastomosis. The reason why the anastomotic fistula occurred 12 months after TP was unclear. Hypersecretion of gastric acid persists in up to 62% of the patients with gastrinomas after surgical excision of tumors; hence, maintaining a postoperative anti-acid therapy is necessary.^[23]^ Ulcer perforation caused by the hypersecretion of gastric acid might be the cause of the rupture of gastrointestinal anastomosis.

## 4 Conclusion

We reported a case of patient diagnosed with MEN-1 associated gastrinoma and died 14 months after TP. Hypersecretion of gastric acid may persist in patients after complete removal of tumors, and we suggest the need to measure the basal acid output and maintain regular anti-acid therapy in a long-term follow-up, considering the possibility of recurrence even after radical surgical resection.

## Author contributions

**Supervision:** Xu-Bao Liu, Wen Wu Shen.

**Writing – original draft:** Shu Gong, Zhi Li.

**Writing – review & editing:** Xin Wang, Wen Wu Shen.
